# Solitary Rectal Ulcer Syndrome - A Rare Entity in the Pediatric Population

**DOI:** 10.5146/tjpath.2025.13667

**Published:** 2025-01-31

**Authors:** Megha Sawhney, Jyotsna Madan, Devajit Nath, Akanksha Bhatia, Neema Tiwari, Umesh Shukla

**Affiliations:** Department of Pathology, Post Graduate Institute of Child Health, Noida, India

**Keywords:** Solitary rectal ulcer, Histopathology, Pediatric

## Abstract

***Objective: *** To study and correlate the clinicopathological findings of Solitary Rectal Ulcer Syndrome (SRUS) in 10 pediatric patients.

***Material and Methods:*** This study is a retrospective study of patients from January 2017 to June 2024. The clinical records were reviewed for details of the clinical presentation, colonoscopic findings, associated local and systemic diseases, and other investigations.

***Results: *** The mean age of presentation was 10±1 years, and the youngest child was 6 years old. The most common clinical presentation was rectal bleeding and a single ulcer on endoscopy. Histological findings included crypt distortion, crypt branching, and fibromuscular obliteration of the lamina propria. Immunohistochemistry (IHC) for Smooth Muscle Actin (SMA) and special staining with Masson Trichrome (MT) were used to highlight fibromuscular areas whenever in doubt.

***Conclusion:*** The pathogenesis of SRUS is not well understood. It may be associated with chronic mucosal and hypoperfusion-induced ischemic injury to the rectal mucosa due to trauma or increased rectal pressure during straining. Solitary rectal ulcer is a misnomer, as the patient may present with multiple or no ulcers. Endoscopy and histopathology help to diagnose SRUS. Timely and correct diagnosis reduces the morbidity associated with this entity.

## INTRODUCTION

Solitary rectal ulcer syndrome (SRUS) is an uncommon benign disorder of defecation with an estimated incidence of 1 case per 100,000 adults per year, with few pediatric cases noted (1). It shares its clinical and pathological features with benign defecation disorders like rectal prolapse (incidence 20%), proctitis cystica profunda, and inflammatory cloacogenic polyp. Hence, all four entities are collectively grouped as mucosal prolapse syndrome (2).

The syndrome affects men and women equally, and can occur at any age although it typically affects young adults with up to 25% of patients aged over 60 at presentation (1). The most commonly accepted etiopathogenetic mechanism of SRUS is a hypoperfusion-induced chronic mucosal ischemic injury to the rectal mucosa. This is associated with paradoxical contraction of the pelvic floor leading to mucosal prolapse and pressure necrosis of the rectal mucosa (3,4).

Clinically, the patient presents with abdominal pain, constipation, and bleeding per rectum. On endoscopy, the ulcer appears as a shallow-based ulcerating lesion encircled by hyperemic mucosa (5,6). SRUS is a misnomer, as the patient may present with multiple ulcers rather than a solitary ulcer. Sometimes the appearance on endoscopy may be confused with an inflammatory polyp, hyperplastic polyp, or rectal carcinoma, leading to a delay in diagnosis and treatment (7). Sometimes, the patients may be asymptomatic and only diagnosed after being investigated for other pathologies (8).

Here we present the clinicopathological spectrum of 10 pediatric cases.

## MATERIAL and METHODS

This retrospective study was conducted on patients seen between January 2017 and June 2024 in the Department of Pathology, Post Graduate Institute of Child Health, Noida. Departmental records were searched for cases diagnosed as SRUS. The clinical records were reviewed for details of the clinical presentation, colonoscopy findings, associated local and systemic diseases, and other investigations.

**Inclusion criteria:** colonoscopic biopsy of patient aged <18 years with histological diagnosis of SRUS, with or without endoscopic findings.

**Exclusion criteria:** colonoscopic biopsy sent with endoscopic features of solitary ulcer, but no histological features of SRUS seen.

Sections showing features of crypt distortion, surface serration, diamond-shaped crypts, fibromuscular obliteration, and chronic inflammation in the lamina propria. The data was tabulated and analyzed.

## RESULTS

**Figure 1:**
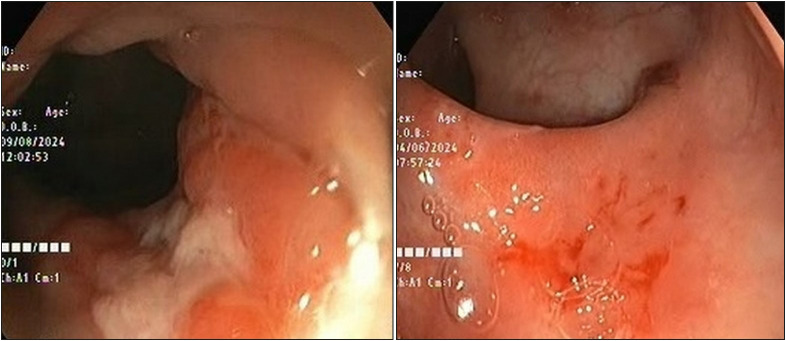
Endoscopy image showing a single ulcer in the left image and mucosal hyperemia in the right image

The male-to-female ratio was 4:1 in the current study (Table 1). The mean age of presentation was 10±1 years, and the most common symptom was rectal bleeding. The second most common presentation symptom was constipation, followed by abdominal pain. The most common endoscopic findings were the presence of a solitary punched-out lesion (single ulcer) 2-3 cm from the anal verge (Figure 1). One case presented with normal endoscopic findings, but the histopathology revealed crypt distortion and fibromuscular infiltration in the lamina propria, leading to the diagnosis of SRUS.

**Table 1:** The clinical and endoscopic findings in our cases

**S. No.**	**Age**	**Sex**	**Clinical presentation**	**Endoscopy findings**
1.	13	M	Rectal bleeding	Single ulcer
2.	8	M	Rectal bleeding, constipation	Single ulcer
3.	12	M	Rectal bleeding	Single ulcer
4.	16	M	Anemia with GI bleed	Multiple ulcers
5.	11	F	Rectal bleeding, constipation	Single ulcer
6.	7	M	Rectal bleeding, constipation, abdominal pain	Normal
7.	9	M	Rectal bleeding, constipation	Single ulcer
8.	8	F	Rectal bleeding, constipation, pain in the abdomen, mucoid discharge	Multiple ulcers
9.	10	M	Rectal bleeding	Single ulcer
10.	6	M	Rectal bleeding, constipation	Single ulcer
Total				Single ulcer: n=7 (70%). Multiple ulcer: n=2 (20%). Normal: n=1 (10%).

**Figure 2:**
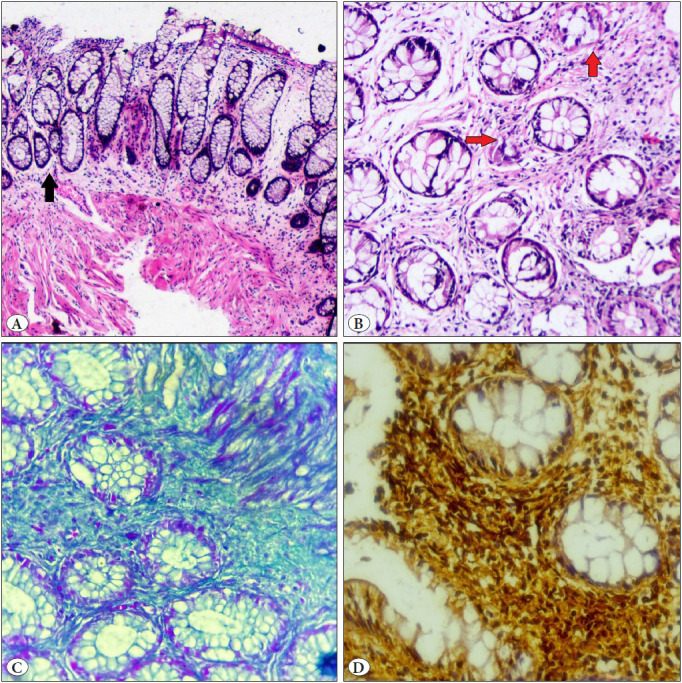
**A)** Section showing fibromuscular obliteration of the lamina propria, loss of surface epithelium, and diamond-shaped crypts (black arrow) (H and E, 200X) **B)** Section showing mild crypt distortion (red arrow) and fibromuscular hyperplasia (H&E, 400X) **C)** Section highlighting fibroblastic area (MT, 400X). **D)** Immunohistochemistry (IHC) for SMA (smooth muscle actin) found to be positive in the fibromuscular areas between the crypts (IHC, 400X).

Table 2 shows the histopathological features of all cases. All cases showed the presence of fibromuscular obliteration of the lamina propria (Figure 2) and chronic inflammation, followed by crypt distortion (Figure 2, red arrow) and surface ulceration. Formation of diamond-shaped crypts was seen in 5 cases (50%) (Figure 2, black arrow). Masson trichrome was used to highlight the fibroblastic area in some cases (Figure 2). Immunohistochemistry (IHC) for SMA (smooth muscle actin) was seen to be positive in the fibromuscular areas between the crypts (Figure 2).

**Table 2:** Histopathological features of all cases

**Histological features**	**Number of cases**	**Percentage**
Crypt distortion	9	90
Surface serration	9	90
Diamond crypts	5	50
Crypt branching	8	80
Fibromuscular obliteration	10	100
Chronic Inflammation in the lamina	10	100

## DISCUSSION

SRUS is a misnomer, as the patient may present without an ulcer, the ulcers may be multiple, or they may not be in the rectum only (9). It is a rare but well-known entity in adults, and a lack of awareness may lead to underdiagnosis or misdiagnosis in children.

The pathogenesis of SRUS is based on theories expressing direct trauma, local ischemia, and paradoxical contraction of pelvic floor muscles. It is hypothesized that straining or sometimes self-induced rectal digitation to remove impacted stool may cause damage to the rectal mucosa (5,10). Another theory states that uncoordinated muscle contraction in the puborectalis muscle will lead to increased pressure in the rectum and anal canal, resulting in ischemia and ulceration (10). In some cases, rectal prolapse and intussusceptions have been associated with local trauma and subsequent ulceration (11).

In the current study, the youngest case was 6 years old, and the oldest patient was 16 years. In a study by Suresh et al., the youngest patient with SRUS was a child of 1.5 years (12). Our study showed that most cases were aged 6 to 10 years. In the largest study in pediatric patients comprising 140 cases, most cases were in the 11-15 years age group followed by the 6-10 years age group (13). Our study showed male preponderance, which is in concordance with studies by Poddar et al., Dehghani et al., and Al-Brahim et al. (4,13,14).

The most common presentation was bleeding per rectum followed by constipation. This is in concordance with studies by Suresh et al. and Al-Brahim et al. (4,12). Although constipation is a frequent symptom of presentation, diarrhea has also been reported in 22% by Torres et al. and in one case by Al-Brahim et al. (4,15). There is no possible explanation for this presentation, but the clinician should be aware of this and consider SRUS as a differential diagnosis even in cases with diarrhea. A study by Gabra et al. has reported a case of SRUS who presented with severe rectal stricture (16). Such clinical presentations and rectal ulcers on endoscopy may direct clinicians toward the diagnosis of carcinoma.

Endoscopic findings in our study showed 7 cases with single ulcers, 2 cases with multiple ulcers, and a single case with normal endoscopy findings. Overall, 90% of the cases showed ulcers, mostly solitary (70%), and all were in the rectum in the current study. This was in concordance with a study on 21 children by Suresh et al. (12). Similarly, Kowalsk-Duplaga et al., in their study of 31 children, showed ulcers in 97% of the cases, out of which 61% were solitary (17). In a study by Abid et al. in 116 SRUS cases, 25% of the cases had polypoidal or nodular growth on endoscopy, 3% erythematous mucosa, and one patient telangiectatic spots (2). Hence, the variability with which SRUS presents on endoscopy is more profound than is generally comprehended.

The most common differential diagnosis of SRUS on endoscopy findings is either IBD (inflammatory bowel disease) or carcinoma (18). One case reported by Bhusal et al. was diagnosed as carcinoma on clinical presentation of bleeding per rectum and broad-based ulcer on endoscopy. In this case, histopathology proved to be a saving tool, and a diagnosis of SRUS was made (19). Characteristic histopathological findings of SRUS include fibromuscular obliteration of the lamina propria, hypertrophied muscularis mucosa with extension of muscle fibers upward (between the crypts), and glandular crypt abnormalities (6). Other findings, such as surface erosions, mild inflammation, distorted crypts, and reactive epithelial atypia, may also be seen in IBD and cancer. IBD cases will also show granuloma formation and involvement of muscularis propria in Crohn’s disease (CD), whereas cryptitis, crypt abscess, and crypt distortion are seen in ulcerative colitis (UC) cases. On colonoscopy, even CD patients may show a cobblestone appearance of mucosa and pseudopolyps as seen in UC.

The treatment options available are suboptimal, and the outcomes may be unsatisfactory despite a correct diagnosis. The treatment protocol involves conservative management that includes patient education and behavioral modification as the first step, followed by a high-fiber diet and bulking laxatives, followed by topical treatments with salicylate, sulfasalazine, steroids, sucralfate, and lastly surgery. Surgery is indicated in failure of conservative treatment to control severe symptoms such as persistent bleeding. Surgical treatments include rectal prolapse correction, ulcer excision, and, rarely, colostomy (13,14). In a study by Abusharifah et al. in 2021, the majority of patients received mesalamine and sucralfate enema as treatment and were reported to show relief in symptoms (20). In their study, around 10% of the children received stool softeners as the sole treatment option.

In a study by Li and Hamilton, it was documented that malignant tumors might present with SRUS-like features on biopsy initially but later develop malignancy (21). This suggests the potential of SRUS to progress to carcinoma. A case of well-differentiated adenocarcinoma in the focus of SRUS has been reported by Tsuchida et al., and it has been speculated that there is a chance of adenocarcinoma originating from the SRUS mucosa (22). Loss of hMLH1 gene expression is seen in many cases of SRUS, again indicating its malignant potential (23).

Although the sample size of the current study is small, this paper throws light on the clinical, endoscopic, and histopathological characteristics of this rare entity in the pediatric population. The condition is benign but the morbidity associated with it in the form of rectal bleeds leading to anemia emphasizes the point of early and timely diagnosis.

## CONCLUSION

Solitary rectal ulcer is a misnomer and clinically mimics IBD and carcinoma. Endoscopy and histopathology help in diagnosing SRUS. Fibromuscular splaying between crypts is the most characteristic finding in the histopathology of SRUS. Timely and correct diagnosis reduces the morbidity associated with this entity. More studies are needed to understand the treatment modality.

## Conflict of Interest

The authors have no conflicts of interest to declare.
